# Choosing among the long-acting injectable antipsychotics: an evidence-based pragmatic guide

**DOI:** 10.1017/S1092852925100400

**Published:** 2025-07-18

**Authors:** Leslie Citrome

**Affiliations:** Department of Psychiatry and Behavioral Sciences, New York Medical College, Valhalla, NY, USA

**Keywords:** Depot, long-acting injectable antipsychotic, schizophrenia, schizoaffective disorder, bipolar disorder, risperidone, paliperidone, aripiprazole, olanzapine

## Abstract

In this review, the aim is to differentiate between the 3 second-generation antipsychotics available as long-acting injectables (risperidone/paliperidone, aripiprazole, and olanzapine) and their varied formulations. Differences and similarities among the available products are discussed, including the amenities of care: route of administration (intramuscular or subcutaneous), injection frequency, needle gauge and length, injection volume, injection site, reconstitution procedures, initiation with oral medication or multiple injections, refrigeration requirements, post-injection observation requirements, drug–drug interactions preventing use or requiring dosing adjustments, adjustments requirements for late or missed doses, availability of patient assistance programs, and access barriers for off-label use. Effectiveness in acute and maintenance treatment are reviewed using the metrics of number needed to treat and number needed to harm.

## Introduction

The availability of long-acting injectable antipsychotics (LAIs) has transformed care for many individuals with schizophrenia, schizoaffective disorder, and bipolar disorder. The “guaranteed delivery” of a medication in a predictable and steady way can decrease the risk of relapse, rehospitalization, as well as reduce mortality.^1^ ^,^^2^ The revised American Psychiatric Association guidelines for the care of people with schizophrenia call out that LAIs should be considered in instances of poor or uncertain adherence, as well as for patients who would prefer this modality.^3^ The guidelines further add that LAIs can be useful in the transition from inpatient to outpatient care. Research has also demonstrated better outcomes when LAIs are used early in the disease course.^4^ ^–^^6^ An important benefit for providers of care is that LAI antipsychotics can eliminate the guesswork about adherence status and allow the clinician to focus on other reasons why symptoms may be exacerbated, such as psychosocial stressors or substance use. In the end, “preventing a relapse today can make a difference for a lifetime.”^7^

Of pragmatic concern is the limited array of molecules available as an LAI formulation. However, the formulations themselves differ, including several that contain the same molecule (Tables 1 and 2). This article reviews the evidence regarding the agents available as LAIs and considerations when selecting one over the other.

**Table 1.** Antipsychotic Molecules with Long-Acting Formulations Available/Approved in the United States as of April 2025

First-generation antipsychotics
• Haloperidol
• Fluphenazine
Second-generation antipsychotics
• Risperidone/paliperidone
• Aripiprazole
• Olanzapine

**Table 2.** Formulations of Long-Acting Injectable Antipsychotics Approved in the United States as of April 2025

First-generation antipsychotics
• Haloperidol decanoate
• Fluphenazine decanoate
Second-generation antipsychotics
• Risperidone- or paliperidone-containing formulations
• Risperidone microspheres (2 products, each administered every 2 wk)
• Risperidone subcutaneous (2 products, 1 administered every month,^a^ the other every month or every 2 mo)
• Risperidone ISM^a^
• Paliperidone palmitate (2 products, 1 administered every month, the other administered every month, every 3 mo, or every 6 mo)
• Aripiprazole-containing formulations
• Aripiprazole monohydrate (administered every month or every 2 mo)
• Aripiprazole lauroxil (administered every month, every 6 wk, or every 2 mo)
• Olanzapine pamoate (administered every 2 wk or every month)

a Discontinued or never commercialized in the United States.

## What are the molecules available as long-acting injectable antipsychotics?

Both first- (typical) and second- (atypical) generation antipsychotics are available as LAI formulations. However, the choice among molecules is currently limited to very few when compared to the plethora of oral treatments available for schizophrenia and bipolar disorder. In the United States, the clinician can choose among haloperidol, fluphenazine, risperidone/paliperidone, aripiprazole, and olanzapine.^7^ Haloperidol and fluphenazine are first-generation antipsychotics and are often co-administered with anticholinergic agents in order to prophylactically address drug-induced parkinsonism and acute dystonia, as well as actively treat these adverse effects when they occur. Not only does this strategy increase pill burden, but anticholinergic load is also associated with cognitive impairment.^8^ ^–^^10^ Moreover, risk for developing tardive dyskinesia is greater for first-generation than for second-generation antipsychotics.^11^ ^,^^12^ Other first-generation LAI antipsychotics have been available outside the United States (eg, flupentixol, perphenazine, pipotiazine, and zuclopenthixol).^13^

Drug-induced parkinsonism and the need for anticholinergic medications can be minimized by using second-generation LAI antipsychotics. Among the second-generation antipsychotics, the molecules available in the United States include risperidone/paliperidone, aripiprazole, and olanzapine. These second-generation antipsychotics appear similar in terms of efficacy, with the possible exception of olanzapine, which may be more efficacious.^14^ ^,^^15^ Of note is that paliperidone is the principal active metabolite of risperidone and shares with it many of its characteristics.^16^ There is more heterogeneity regarding tolerability than with efficacy,^17^ with liability for weight gain and metabolic adverse effects being more prominent for olanzapine, followed by risperidone, and aripiprazole; prolactin elevation with risperidone/paliperidone, followed by olanzapine, and aripiprazole; and somnolence with olanzapine, followed by risperidone, and aripiprazole.

A major treatment conundrum is the prescribing of an oral medication that appears efficacious and tolerable, but that does not have a corresponding long-acting formulation. Considerations when switching to an LAI antipsychotic will include the uncertainty of adequate response to a different molecule delivered by injection, balanced by the issues around poor or uncertain adherence with an oral agent. A not uncommon scenario is the patient who appears not to respond to oral antipsychotics and is thus considered potentially “treatment-resistant”.^18^ Care should be taken to rule out pseudo-resistance, which can confound the assessment of a patient who is covertly nonadherent, in which case a trial of a LAI antipsychotic is recommended to determine if the person is truly treatment resistant.^19^

A complete discussion of the similarities and differences among the oral formulations of risperidone/paliperidone, aripiprazole, and olanzapine (and others) is beyond the scope of this review. The reader is referred to a number of meta-analyses and other reviews that serve to compare and contrast,^17^ ^,^^20^ as well as indirect comparisons using the metrics of number needed to treat and number needed to harm (Ref.,^21^ and also see Figure 1 and Table 6 as published in Ref. ^22^).

## What are the formulations available?

Older first-generation antipsychotics such as fluphenazine decanoate (administered generally every 2 weeks) and haloperidol decanoate (administered generally every 4 weeks) remain available.^23^ They are relatively inexpensive, and haloperidol decanoate remains frequently prescribed. Both fluphenazine decanoate and haloperidol decanoate can be injected in either the deltoid or gluteal muscle, and fluphenazine decanoate can also be injected subcutaneously, although this is not commonly done.^24^ The first-generation antipsychotic LAIs are dissolved in sesame seed oil and can be more challenging to inject than the second-generation intramuscular LAI formulations, which are all suspended in water.

For fluphenazine decanoate, the initial dose is 12.5–25 mg and can be increased in increments of 12.5 mg, but the total amount administered at one time should not exceed 100 mg.^23^ ^,^^24^ The initial dose for haloperidol decanoate should be 10–20 times the previous daily dose in oral haloperidol equivalents, but the initial injection is limited to 100 mg, followed by the balance 3–7 days later. The usual maintenance range is 10–15 times the previous daily dose in oral haloperidol equivalents, depending on clinical response.^23^ ^,^^25^ Clinical experience with haloperidol decanoate at doses greater than 450 mg/month is limited.^25^ In order to reduce injection volume, a more concentrated formulation (100 mg/ml versus 50 mg/ml) is available.^25^

Among the 3 different second-generation antipsychotics currently available in LAI formulations, risperidone/paliperidone, aripiprazole, and olanzapine, several choices are currently available for risperidone/paliperidone and aripiprazole, and currently only 1 for olanzapine, although a subcutaneous formulation is in the late stage of development.^26^ The different formulations containing the same active molecule can be differentiated in terms of their “amenities of care” such as dosing intervals (from every 2 weeks to every 6 months), availability of different dose strengths, choice of injection site (deltoid muscle, gluteal muscle, subcutaneous), gauge and length of the needle, injection volume, storage and reconstitution requirements, need for oral supplementation, guidance regarding early or late dosing, approved indication, and requirement for observation post injection.^7^ A list of these pragmatic considerations is contained in Table 3.

**Table 3.** Long-Acting Injectable Antipsychotics: “Amenities of Care” (Adapted from Ref.^7^)

Is this injection intramuscular or subcutaneous?
How often are the injections administered?
What is the needle gauge?
What is the injection volume?
Is there a choice of injection site?
Does this product require reconstitution?
Is oral supplementation required?
Does storage of this product require refrigeration?
Are there any special requirements for post-injection observation?
Are there any important drug–drug interactions, and can they be remedied?
Missed doses: What is the “grace period?”
Is reimbursement an issue if used “off-label”?
In case of reimbursement obstacles, can I easily access a patient-assistance program?

## Long-acting injectable risperidone and paliperidone formulations, as approved by the US Food and Drug Administration

Table 4 outlines the 9 different formulations approved by the US Food and Drug Administration (FDA) that contain risperidone or paliperidone (2 are either discontinued or never commercialized in the United States). They differ broadly in approved indications, dosage forms/strengths, reconstitution requirements, injection sites and method of injection (intramuscular versus subcutaneous), needle gauge/length, injection volume, injection interval, requirement for oral supplementation, need for refrigeration when stored, and instructions for early or late dosing.

**Table 4.** Long-Acting Injectable Risperidone and Paliperidone Formulations, as Approved by the US Food and Drug Administration (Adapted from Table 2 in Faden J, Citrome L. A new paliperidone palmitate formulation: how is it different and where does it fit in our array of choices for long-acting formulations of risperidone and paliperidone? *Curr Med Res Opin.* 2025;41(4):663–666. doi:10.1080/03007995.2025.2482654. Open Access distributed under the terms of the Creative Commons Attribution-NonCommercial-NoDerivatives License (http://creativecommons.org/licenses/by-nc-nd/4.0/)

Formulation (manufacturer)	Risperidone IM 2 wk (Janssen)	Risperidone IM 2 wk (Luye)	Risperidone SC monthly (Indivior)	Risperidone SC monthly or every 2 mo (Teva)	Risperidone ISM (Rovi)	Paliperidone palmitate (Luye)	Paliperidone palmitate (Janssen)	Paliperidone palmitate-3 Mo (Janssen)	Paliperidone palmitate-6 Mo (Janssen)
Brand name (United States)	Risperdal Consta	Rykindo	Perseris	Uzedy	Risvan	Erzofri	Invega Sustenna	Invega Trinza	Invega Hayfera
Year approved	2003	2023	2018	2023	2024	2024	2009	2015	2021
Approved indications	Schizophrenia, maintenance of bipolar I disorder (monotherapy or adjunct to lithium/valproate)	Schizophrenia, maintenance of bipolar I disorder (monotherapy or adjunct to lithium/valproate)	Schizophrenia	Schizophrenia	Schizophrenia	Schizophrenia, Schizoaffective disorder	Schizophrenia, Schizoaffective disorder	Schizophrenia	Schizophrenia
Basis for FDA approval (study type)	One RCT in patients with schizophrenia (acute), 1 RCT maintenance monotherapy in patients with bipolar I disorder, and one RCT maintenance adjunctive therapy in patients with bipolar I disorder	Pharmacokinetic studies	One phase 3, RCT (acute)	One phase 3, RCT (maintenance)	One phase 3, RCT (acute)	Pharmacokinetic studies	Four short-term monotherapy, I maintenance monotherapy (Schizophrenia); maintenance treatment as monotherapy and as adjunct to mood stabilizer or antidepressant (Schizoaffective disorder)	One long-term, phase 3, RCT	One long-term, phase 3, RCT
Injection intervals	2 wk	2 wk	1 mo	1 mo, 2 mo	1 mo	1 mo	1 mo	3 mo	6 mo
Injection type, sites	IM, deltoid. gluteal	IM, gluteal	SC, abdomen, upper arm	SC, abdomen, upper arm	IM, deltoid, gluteal	IM, deltoid for initiation dose, deltoid or gluteal for maintenance dose	IM, deltoid for initiation dose, deltoid or gluteal for maintenance dose	IM, deltoid, gluteal	IM, gluteal
Number of injections necessary at start	1	1	1	1	1	1	2	1 (after at least 4 mo on monthly paliperidone palmitate)	1 (after at least 4 mo of monthly paliperidone palmitate or after receiving paliperidone palmitate–3 mo)
Needle gauge and length	21G/1-inch deltoid; 20G/2-inch gluteal	20G/2-inch	18G/⅝-inch	21G/⅝-inch	21G/1-inch deltoid; 20G/2-inch gluteal	For deltoid muscle, 23G/1-inch if patient is <90 kg, and 22G/1 ½-inch, if patient is ≥90 kg; for gluteal muscle injections, 22G/1 ½-inch	For deltoid muscle, 23G/1-inch if patient is <90 kg, and 22G/1 ½-inch, if patient is ≥90 kg; for gluteal muscle injections, 22G/1 ½-inch	For deltoid muscle, 22G/1-inch if patient is <90 kg, and 22G/1 ½-inch, if patient is ≥90 kg; for gluteal muscle injections, 22G/1 ½-inch	20G/1 ½ –inch
Injection volume	2 mL (all doses)	2 mL (all doses)	0.6 mL (90 mg), 0.8 mL (120 mg)	0.14 mL (50 mg), 0.21 mL (75 mg), 0.28 mL (100 mg), 0.35 mL (125 mg), 0.42 mL (150 mg), 0.56 mL (200 mg), 0.7 mL (250 mg)	0.55 mL (75 mg), 0.73 mL (100 mg)	0.25 mL (39 mg), 0.5 mL (78 mg), 0.75 mL (117 mg), 1.0 mL (156 mg), 1.5 mL (234 mg), 2.25 mL (351 mg)	0.25 mL (39 mg), 0.5 mL (78 mg), 0.75 mL (117 mg), 1.5 mL (156 or 234 mg)	0.875 mL (273 mg), 1.32 mL (410 mg), 1.75 mL (546 mg), 2.63 mL (819 mg)	3.5 mL (1092 mg), 5 mL (1560 mg)
Oral supplementation	21 d	7 d	None	None	None	None	None	None	None
Length of time it can be kept at room temperature rather than refrigerated	7 d	7 d	30 d	90 d	NA—always stored at room temperature	NA—always stored at room temperature	NA—always stored at room temperature	NA—always stored at room temperature	NA—always stored at room temperature
Reconstitution required	Yes (combine the powder in the vial with the diluent provided in the syringe)	Yes (combine the powder in the vial with the diluent provided in the syringe)	Yes (place the liquid syringe on top of the powder syringe [to prevent powder spillage] and connect the syringes and push back and forth for 60 cycles)	No reconstitution required; ready to use after whipping the prefilled syringe 3 times	Yes (place the solvent syringe on top of the powder syringe [to prevent powder spillage] and connect the syringes and push back and forth for 50 cycles)	No reconstitution required; ready to use after shaking the prefilled syringe vigorously for a minimum of 10 s	No reconstitution required; ready to use after shaking the prefilled syringe vigorously for a minimum of 10 s	No reconstitution required; ready to use after shaking the prefilled syringe vigorously for a minimum of 15 s	No reconstitution required; ready to use after shaking the prefilled syringe vigorously for a minimum of 15 s, resting briefly, then shaking again for a minimum of 15 s
Doses available	12.5, 25, 37.5, 50 mg (there is no dose equivalency with oral risperidone documented in product labeling)^c^	12.5, 25, 37.5, 50 mg (there is no dose equivalency with oral risperidone documented in product labeling)^c^	90, 120 mg (equivalent to oral risperidone 3 mg and 4 mg/d, respectively)	50 mg, 70 mg, 100 mg, 125 mg, 150 mg, 200 mg, 250 mg (equivalent to oral risperidone 2 mg to 5 mg/d)	75 mg, 100 mg (equivalent to oral risperidone 3 mg and 4 mg/d, respectively)	39 mg, 78 mg, 117 mg, 156 mg, 234 mg, 351 mg	39 mg, 78 mg, 117 mg, 156 mg, 234 mg	273 mg, 410 mg, 546 mg, 819 mg	1092 mg, 1560 mg

Abbreviations. IM, intramuscular; SC, subcutaneous; RCT, randomized controlled trial.

a On July 9, 2024, the manufacturer announced the cessation of all sales and marketing activities regarding this product, but will continue to supply it for the foreseeable future to avoid disruption to patient care (see https://otp.tools.investis.com/clients/uk/indivior2/ms/regulatory-story.aspx?newsid=1 840 448).

b On November 7, 2024, the manufacturer announced that the product will not be marketed in the United States and will focus on European development (https://www.rovi.es/en/content/first-nine-months-2024-results-press-release).

c Product labeling states that the recommended dose for the treatment of schizophrenia is 25 mg IM Q2W; patients not responding to 25 mg may benefit from 37.5 mg or 50 mg.

*Source*: Adapted from Faden et al.^[44]^

In 2003, risperidone microspheres (Risperdal Consta, Janssen) became the first second-generation antipsychotic to be available in a LAI formulation at doses of 12.5, 25, 37.5, or 50 mg, administered every 2 weeks.^27^ ^,^^28^ In addition to the indication for schizophrenia, in 2009, risperidone microspheres was also approved as monotherapy or as adjunctive therapy to lithium or valproate for the maintenance treatment of bipolar I disorder.^29^ ^,^^30^ Of note, storage of the product requires refrigeration, but it can remain at room temperature for up to 7 days.^30^ Because the main release of the drug does not begin until 3 weeks after administration, supplemental oral risperidone is required for 21 days after the first injection (hence overlapping the second injection) and after any dose increase.^30^ After mixing the risperidone microspheres powder with the supplied aqueous diluent, it can be administered in the deltoid or gluteal muscle. The recommended starting dose is 25 mg/2 weeks, and the maximum recommended dose is 50 mg/2 weeks. With respect to total exposure, injections of 25, 50, or 75 mg every 2 weeks were found to be equivalent to daily oral doses of 2, 4, or 6 mg of risperidone, respectively.^31^

In 2023, another formulation of risperidone microspheres (Rykindo, Luye) from a different pharmaceutical company was approved by the FDA for the same indications as the original.^32^ Approval was based on a pharmacokinetic study that demonstrated similar plasma levels as the original product.^33^ Although both formulations of risperidone microspheres share many common characteristics, they do differ in their pharmacokinetic profile. This newer version requires only 1 week of oral supplementation and is currently only approved for gluteal injection. The dosing recommendations for the 2 risperidone microsphere products are otherwise identical, save for the original formulation, where upward dose adjustment of risperidone microspheres (Janssen) should not be made more frequently than every 4 weeks and that the clinical effects of this dose adjustment should not be anticipated earlier than 3 weeks after the first injection with the higher dose.^30^ When switching to the newer formulation (Luye), the dose should be the same as that of the previous treatment, with the first injection given 4 weeks (no later than 5 weeks) after the last injection of the previous treatment.^32^

Three more novel risperidone formulations have been approved by the FDA for the treatment of schizophrenia, and importantly, do not require oral supplementation upon initiation: a subcutaneous preparation^34^ that can be administered monthly but requires reconstitution (Perseris, Indivior; approved in 2018 and now discontinued^35^ and thus not described here in detail), a subcutaneous preparation (Uzedy, Teva, referred to later as 1-month/2-month risperidone subcutaneous) that comes in a prefilled syringe and that can be administered on Day 1 either every month or every 2 months (approved in 2023,^36^ and described below), and an intramuscular formulation that requires reconstitution and can be administered monthly (Risvan, Rovi); although FDA approved in 2024, this formulation is currently not anticipated to be available commercially in the United States and thus not described here in detail.^33^ ^,^^37^

One-month/2-month risperidone subcutaneous is available to match oral risperidone doses of 2, 3, 4, and 5 mg/d, with once monthly 50, 75, 100, and 125 mg, and 2-month 100, 150, 200, and 250 mg, respectively.^36^ Oral supplementation or a loading dose is not required upon initiation at any dose. This formulation can be stored at room temperature for up to 90 days; otherwise, it must be refrigerated.^36^

The product labels for the risperidone formulations contain similar advice that a dose adjustment may be necessary in the presence of drug–drug interaction(s) with CYP2D6 and/or CYP3A4 inhibitors and/or if the patient is a CYP2D6 poor metabolizer, or if the patient is taking a CYP3A4 inducer.^30^ ^,^^32^ ^,^^34^ ^,^^36^ ^,^^37^

Paliperidone (9-OH risperidone) is the main active metabolite of risperidone, and a once-monthly injectable formulation (Invega Sustenna, Janssen) became available in the United States in 2009.^38^ ^–^^40^ In contrast to risperidone microspheres, which must be reconstituted as well as stored in a refrigerator, paliperidone palmitate is an aqueous suspension that comes in prefilled syringes and does not require refrigeration. Paliperidone palmitate has relatively small needle bores to select from. Instead of using oral supplementation, the initiating doses are all by injection: 234 mg on treatment day 1 and 156 mg 1 week later (± 4 days), both administered in the deltoid muscle. Although the recommended monthly maintenance dose is 117 mg for the treatment of schizophrenia, the maintenance dose can be within the range of 39–234 mg, equivalent to the dose range of 3–12 mg/d for oral paliperidone.^38^ When converting from oral risperidone to paliperidone palmitate, oral risperidone doses of 1, 2, 3, 4, and 6 mg/d result in similar exposures as 39, 78, 117, 156, and 234 mg of paliperidone palmitate, respectively.^41^ The regular monthly maintenance doses can be administered in either the deltoid or the gluteal muscle. The product label recommends avoiding the use of a strong inducer of CYP3A4 and/or P-glycoprotein, such as carbamazepine, during a dosing interval. In addition to the indication for treatment of schizophrenia, paliperidone palmitate once monthly received approval in 2014 for use in schizoaffective disorder as monotherapy or as an adjunct to mood stabilizers or antidepressants.^29^ ^,^^40^ ^,^^42^

In 2024, a new formulation of paliperidone palmitate (Erzofri, Luye) was approved in the United States for the treatment of schizophrenia and schizoaffective disorder^43^ ^,^^44^ and differs from the original in that it can be initiated with a single 351 mg dose administered in the deltoid muscle. Approval was based on a pharmacokinetic study,^44^ and this new formulation carries the identical dosing recommendations after initiation as the original.^38^ ^,^^43^

An extension to the monthly injection interval became available in 2015 in the form of a 3-month formulation of paliperidone palmitate (Invega Trinza, Janssen). The indication is restricted to schizophrenia and for individuals who have been treated with the once-monthly formulation of paliperidone palmitate for ≥4 months.^29^ ^,^^45^ The 3-month formulation is packaged in water-based prefilled syringes; however, the product is denser than the once-monthly formulation and has a larger particle size.^46^ The doses that are available remain sufficiently small in volume so that they can be administered in the deltoid muscle, although gluteal injection remains an option. Dose for the 3-month formulation is calculated by multiplying the once-monthly dose by 3.5, and is available for the dose equivalent of 78, 117, 156, and 234 mg of once-monthly paliperidone palmitate. The 3-month formulation requires the use of special-purpose thin-walled needles that come packaged with the product, and these needles cannot be interchanged with those supplied with the once-monthly formulation or with other regular commercially available needles. A 6-month version of paliperidone palmitate (Invega Hafyera, Janssen) was approved in 2021^47^ ^,^^48^ with doses of 1092 and 1560 mg, equivalent to 156 and 234 mg of the once-monthly formulation, and 546 and 819 mg of the 3-month formulation, respectively. Similar to the 3-month formulation, the 6-month formulation is restricted to schizophrenia and for individuals who have been treated with the once-monthly formulation of paliperidone palmitate for ≥4 months, although it is possible to go from the 3-month to the 6-month formulation at the time of the next scheduled injection.^47^

## Long-acting injectable aripiprazole formulations, as approved by the US Food and Drug Administration

Table 5 outlines the 4 different formulations available that contain aripiprazole, with one of them (aripiprazole lauroxil nanocrystal dispersion) reserved for the initiation of aripiprazole lauroxil.^29^ ^,^^49^ ^–^^56^ Principal differences between the formulations include approved indications, dosage forms/strengths, reconstitution requirements, injection sites, needle gauge/length, injection volume, injection interval, requirement for oral supplementation, concomitant use instructions with CYP3A4 inducers, and instructions for early or late dosing.

**Table 5.** Long-Acting Injectable Aripiprazole Formulations, as Approved by the US Food and Drug Administration (Adapted from Ref.^7^)

	Aripiprazole monohydrate monthly (Abilify Maintena)	Aripiprazole monohydrate 2 mo (Abilify Asimtufii)	Aripiprazole lauroxil (Aristada)	Aripiprazole lauroxil Nanocrystal dispersion (Aristada Initio)
Year approved	2013	2023	2015	2018
Approved indications (all adult)	Schizophrenia; bipolar I disorder maintenance treatment (monotherapy) (2017)	Schizophrenia; bipolar I disorder maintenance treatment (monotherapy)	Schizophrenia	Schizophrenia
Dosage forms/strengths	Vial kits and dual-chambered prefilled syringes: 300 mg, 400 mg	Injectable suspension: 720 mg, 960 mg	Injectable suspension: 441 mg, 662 mg, 882 mg, 1064 mg	Injectable suspension: 675 mg
Requires adding diluent/liquid	Yes	No	No	No
Injection type	Intramuscular	Intramuscular	Intramuscular	Intramuscular
Approved injection sites	Deltoid or gluteal muscle	Gluteal muscle	Deltoid (441 mg only) or gluteal muscle	Deltoid or gluteal muscle
Needle gauge and length	21G/2-inch, 22G/1.5-inch, or 23G/1-inch	21G/2-inch, 22G/1.5-inch	20G/1.5 or 2-inch, 21G/1-inch	20G/1.5 or 2-inch, 21G/1-inch
Injection volume	200 mg/mL; range 0.8 mL (160 mg) to 2 mL (400 mg)	2.4 mL (720 mg); 3.2 mL (960 mg)	276 mg/mL: range 1.6 mL (441 mg) to 3.9 mL (1064 mg)	2.4 mL
Injection interval (weeks)	4	8	4, 6, or 8	Not applicable
Starting dose	400 mg	960 mg	441 mg/4 wk, 662 mg/4 wk, 882 mg/4 wk, 882 mg/6 wk, 1064 mg/8 wk	Not applicable
Maintenance dose	300 or 400 mg/4 wk (adjust for CYP2D6 or CYP3A4 inhibitors; cannot give with CYP3A4 inducers)	720 or 960 mg/8 wk (adjust for CYP2D6 or CYP3A4 inhibitors; cannot give with CYP3A4 inducers)	Same as above (adjust for CYP2D6 or CYP3A4 modulators)	Not applicable
Oral dose equivalent	Blood levels achieved with aripiprazole monohydrate 400 mg are comparable with those of oral aripiprazole 15 to 20 mg/d	Blood levels achieved with aripiprazole monohydrate 960 mg are comparable with those of oral aripiprazole 15 to 20 mg/d	Aripiprazole lauroxil 441 mg monthly is equivalent to oral aripiprazole 10 mg/d; 662 mg monthly, 882 mg every 6 wk, or 1064 mg every 2 mo is equivalent to oral aripiprazole 15 mg/d; 882 mg monthly is equivalent to oral aripiprazole ≥20 mg/d	Not applicable
Oral supplementation	14 d after the initial injection or 1 d when used with an additional IM dose of 400 mg and one 20 mg dose of oral aripiprazole	14 d after the initial injection or 1 d when used with an additional IM dose of 400 mg and one 20 mg dose of oral aripiprazole	21 d after the initial injection or 1 d when used with the nano-crystal dispersion formulation and one 30 mg dose of oral aripiprazole	One d of aripiprazole 30 mg
Stored refrigerated	No	No	No	No

Aripiprazole monohydrate (Abilify Maintena, Otsuka/Lundbeck) was approved in the United States in 2013.^23^ ^,^^29^ ^,^^49^ ^,^^52^ In addition to being approved for the treatment of schizophrenia, aripiprazole monohydrate was approved in 2017 for maintenance monotherapy treatment of bipolar I disorder.^29^ ^,^^52^ Following reconstitution with water using either a 300 or 400 mg vial kit for doses as low as 160 mg, or prefilled dual-chambered syringes for the 300 and 400 mg dose strengths, the monthly injection can be administered in either the deltoid or gluteal muscle. The recommended initial and maintenance doses are 400 mg, although a reduction to 300 mg can be considered to manage tolerability concerns. Starting doses of 300, 200, and 160 mg are intended to be used in the presence of drug–drug interaction(s) with CYP2D6 and/or CYP3A4 inhibitors and/or if the patient is a CYP2D6 poor metabolizer. Use is to be avoided in the presence of a drug–drug interaction with a CYP3A4 inducer. There are 2 ways to initiate treatment with aripiprazole monohydrate: a 1-day initiation where 2 separate intramuscular injections of 400 mg (or 300 mg if there is a drug–drug interaction with a CYP2D6 or CYP3A4 inhibitor or if the patient is a CYP2D6 poor metabolizer) is administered along with a single oral dose of aripiprazole 20 mg; or a 14-day initiation where in conjunction with the first administration of aripiprazole monohydrate 400 mg (or 300 mg if there is a drug–drug interaction with a CYP2D6 or CYP3A4 inhibitor or if the patient is a CYP2D6 poor metabolizer), 14 consecutive days is required of either oral aripiprazole (10–20 mg) or the current oral antipsychotic. Blood levels achieved with aripiprazole monohydrate 400 mg are comparable with those of oral aripiprazole 15–20 mg/d.^57^ The 1-day initiation regimen was approved by the FDA in 2025, after having been available in Europe since 2020.^58^

In 2023, a 2-month formulation of aripiprazole monohydrate (Abilify Asimtufii, Otsuka/Lundbeck) was approved for the treatment of schizophrenia and for the maintenance treatment of bipolar I disorder.^53^ Approval was based on a pharmacokinetic bridging study comparing the 2-month and once-monthly formulations,^59^ ^–^^61^ and legacy data regarding the once-monthly formulation.^53^ On average, 960 mg of the 2-monthly formulation provides sustained plasma concentrations comparable to 400 mg of the once-monthly formulation, but over a period of 2 months. In contrast to the once-monthly preparation, it is packaged in a ready-to-use prefilled syringe. The available doses of 960 and 720 mg are equivalent to the monthly doses of 400 and 300 mg, respectively. Lower dose strengths are not available. Injection is into the gluteal muscle only.^53^ The 2-month formulation can be started on Day 1 by either administering an additional injection 400 mg (or 300 mg if there is a drug–drug interaction with a CYP2D6 or CYP3A4 inhibitor or if the patient is a CYP2D6 poor metabolizer) of the monthly formulation in a separate gluteal or deltoid muscle along with 1 dose of oral aripiprazole 20 mg; or a 14-day initiation can be used where in conjunction with the first administration of the 2-month formulation, 14 consecutive days is required of either oral aripiprazole (10–20 mg) or the current oral antipsychotic. For patients already receiving the monthly formulation, the 2-month formulation can be given at the time of the next scheduled injection. Of note, the 2-month formulation can be administered within a window of 2 weeks before to 2 weeks after the scheduled date.^53^

Aripiprazole lauroxil (Aristada, Alkermes) is a different aripiprazole LAI formulation that was approved in the United States in 2015.^29^ ^,^^49^ ^,^^54^ ^,^^62^ Aripiprazole lauroxil is supplied in prefilled syringes as an aqueous suspension.^54^ Once injected into the deltoid muscle (approved for the 441 mg dose) or gluteal muscle (approved for any of the doses), the conversion of aripiprazole lauroxil to aripiprazole is governed by the slow dissolution of aripiprazole lauroxil and subsequent enzyme-mediated cleavage by esterases. When the product was launched, dose strengths of 441, 662, and 882 mg were initially available. These doses, when administered monthly, yield exposures to aripiprazole equivalent to oral aripiprazole 10, 15, and ≥ 20 mg/d, respectively. The dose of 882 mg administered every 6 weeks yields similar exposures as 662 mg administered monthly. In 2017, a dose strength of aripiprazole lauroxil 1064 mg administered every 2 months became available and yields equivalent exposures as 662 mg monthly or 882 mg every 6 weeks.^62^ ^,^^63^ Instructions for the use of aripiprazole lauroxil suggest that any dose can be initiated on Day 1, including 1064 mg every 2 months. Selection of a dose is also dependent on the presence of drug–drug interaction(s) with CYP2D6 and/or CYP3A4 inhibitors and/or if the patient is a CYP2D6 poor metabolizer, or if the patient is taking a CYP3A4 inducer.^54^ Initiation of aripiprazole lauroxil requires either 21 days of supplementation with oral aripiprazole or the use of the aripiprazole lauroxil nanocrystal dispersion (ALNCD) formulation (Aristada Initio, Alkermes), available since 2018.^50^ ^,^^51^ ^,^^54^ ^–^^56^ ALNCD contains smaller particles than those used in standard aripiprazole lauroxil, and these particles have faster dissolution properties when injected into the muscle.^51^ An injection of the 675 mg ALNCD formulation into either the deltoid or gluteal muscle, plus administration of oral aripiprazole 30 mg that same day, can substitute for the 21 days of oral supplementation that would otherwise be required upon initiation.^56^ The first injection of standard aripiprazole lauroxil may be administered on the same day as the ALNCD formulation or up to 10 days thereafter.^54^ ^,^^55^ ALNCD is available in only 1 dose strength, thus its use is not possible in the presence of potential drug–drug interactions, such as with strong CYP2D6 or CYP3A4 inhibitors and strong CYP3A4 inducers, or if the patient is a CYP2D6 poor metabolizer.^54^ ^,^^55^

## Long-acting injectable olanzapine formulations, as approved by the US Food and Drug Administration

Table 6 outlines the characteristics of olanzapine pamoate (Zyprexa Relprevv, Eli Lilly).^64^ ^–^^66^ There are currently no alternative LAI formulations of olanzapine commercially available, although a subcutaneous formulation is in late stage of development.^26^ Olanzapine pamoate was approved in the United States in 2009. It differs from the other LAI antipsychotics in that its use is governed by a Risk Evaluation and Mitigation Strategy program (REMS), requiring a 3-hour post-injection monitoring period after each injection.^66^ This is to better manage the potential risk of Post-injection Delirium Sedation Syndrome (PDSS), as described below, a risk that appears to be absent with the experimental subcutaneous formulation.^67^

**Table 6.** Long-Acting Injectable Olanzapine Formulations, as Approved by the US Food and Drug Administration (Adapted from Ref.^7^)

	Olanzapine pamoate (Zyprexa Relprevv)
Year approved	2009
Active moiety	Olanzapine
Approved indications (all adult)	Schizophrenia
Dosage forms/strengths	Vial kits: 210 mg, 300 mg, 405 mg
Requires adding diluent/liquid	Yes
Injection type	Intramuscular
Approved injection sites	Gluteal muscle
Needle gauge and length	19G/1.5-inch
Injection volume	150 mg/mL; range 1.0 mL (150 mg) to 2.7 mL (405 mg)
Injection interval (weeks)	2 or 4
Starting dose	210 mg/2 wk, 405 mg/4 wk, 300 mg/2 wk
Maintenance dose	150 mg/2 wk, 300 mg/4 wk, 210 mg/2 wk, 405 mg/4 wk, 300 mg/2 wk
Oral dose equivalent	For oral olanzapine 10 mg/d, the starting olanzapine pamoate dose is 210 mg every 2 wk or 405 mg every 4 wk, with a maintenance dose of 150 mg every 2 wk or 300 mg every 4 wk; for olanzapine 15 mg/d, the starting olanzapine pamoate dose is 300 mg every 2 wk, with a maintenance dose of 210 mg every 2 wk or 405 mg every 4 wk; for oral olanzapine 20 mg/d, the starting and maintenance dose of olanzapine pamoate is 300 mg every 2 wk
Oral supplementation	No
Stored refrigerated	No
Mandated observation period post-injection	3 h

Olanzapine pamoate is a crystalline salt formulation composed of olanzapine and pamoic acid.^64^ ^,^^65^ After reconstitution in water, it is injected into the gluteal muscle, and the salt slowly dissolves, releasing olanzapine over a period of weeks. However, when olanzapine pamoate comes into contact with a substantial amount of blood or plasma, the salt dissolves more quickly, resulting in the release of a larger amount of olanzapine, potentially leading to PDSS characterized by sedation, confusion, slurred speech, altered gait, or unconsciousness. PDSS can be expected to occur in approximately 0.07% of injections and is time-limited but may require symptomatic treatment.^68^ ^,^^69^ Because there are no clear identifiable risk factors, a REMS was instituted, and olanzapine pamoate can only be provided at registered healthcare facilities, and patients must be monitored by appropriately qualified staff for at least 3 hours after injection.^64^ ^,^^66^ In addition, patients must be accompanied to their next destination upon leaving the facility. Because the risk of PDSS is cumulative, patients receiving olanzapine pamoate every 2 weeks can decrease their risk of PDSS by 50% by switching to monthly injections. PDSS is not common; from a provider perspective, a clinic with 60 patients receiving an injection every 2 weeks might expect approximately 1 event per year.^68^ Although all the other LAI antipsychotics have hypersensitivity listed as a contraindication, no contraindications are noted in the product label for olanzapine pamoate.^64^

Initiation of olanzapine pamoate does not require oral supplementation; however, a higher dose is administered for the first injection, with the exception of the highest dose available.^64^ The starting and maintenance dose is dependent on the dose of oral olanzapine required for stabilization: for patients requiring olanzapine 10 mg/d, the starting olanzapine pamoate dose is 210 mg every 2 weeks or 405 mg every 4 weeks, and then if clinically indicated, patients can be evaluated 2 months later for a reduction to a maintenance dose of 150 mg every 2 weeks or 300 mg every 4 weeks; for patients requiring oral olanzapine 15 mg/d, the starting olanzapine pamoate dose is 300 mg every 2 weeks, and then if clinically indicated, patients can be evaluated 2 months later for a reduction to a maintenance dose of 210 mg every 2 weeks or 405 mg every 4 weeks; for patients requiring oral olanzapine 20 mg/d, the recommended starting and maintenance dose of olanzapine pamoate is 300 mg every 2 weeks.^64^

## Acute treatment with long-acting injectable antipsychotics: what is the evidence?

Among the approved second-generation antipsychotic LAI formulations in the United States, efficacy in acutely exacerbated patients with schizophrenia has been formally evaluated for once-monthly paliperidone palmitate,^70^ ^–^^73^ olanzapine pamoate,^74^ aripiprazole monohydrate once-monthly,^75^ aripiprazole lauroxil,^76^ risperidone subcutaneous injection (Indivior),^77^ and risperidone IM (Rovi).^78^ Although the initiation procedures vary among the different products, starting an LAI antipsychotic while a patient is hospitalized with an acute exacerbation of schizophrenia has consistently demonstrated robust superiority over placebo in reducing psychotic symptoms.^79^ Of additional interest is the ability to reduce hostility and agitation.^80^ ^,^^81^ For LAI formulations not formally assessed in an acute trial, but for which supporting data exists for that molecule, it would be expected that they too would be suitable for acute use (for example, 2-month aripiprazole monohydrate and 1-month/2-month risperidone subcutaneous injection).

Aside from potential adverse effects related to the injection itself (such as pain, redness, induration, or nodule in case of subcutaneous injection), there are adverse reactions related to the molecule itself. Number needed to harm (NNH) versus placebo can be used to indirectly compare risk for weight gain, sedation, and akathisia (Table 7).^30^ ^,^^34^ ^,^^38^ ^,^^52^ ^,^^54^ ^,^^64^ ^,^^78^ ^,^^82^ ^–^^87^ NNH values less than 10 denote events that would be more commonly encountered; this would be the case for weight gain ≥7% from baseline for risperidone subcutaneous injection (Indivior), aripiprazole monohydrate, and olanzapine pamoate, as calculated from their short-term acute registration studies. The weight gain data is counter-intuitive for risperidone subcutaneous injection and aripiprazole monohydrate and appears to differ somewhat from what has been calculated from registration studies of the oral formulations of risperidone and aripiprazole, where the NNH versus placebo estimates for weight gain ≥7% were 18 and 21, respectively^84^ ^,^^86^; this could be a reflection of study design, where patients remained hospitalized throughout the study and potential skewing of the characteristics of the study participants toward those more prone to weight gain.^75^ ^,^^77^ It has also been suggested that the acute use of LAI antipsychotics may be better tolerated than oral formulations.^88^

**Table 7.** Rates and Number Needed to Harm versus Placebo for Weight Gain, Somnolence/Sedation, and Akathisia, for Approved Long-Acting Injectable Second-Generation Antipsychotics and Their Oral Counterparts in Adults as Observed in Acute Short-Term Studies of Long-Acting Injectable Antipsychotics for Schizophrenia (Doses Pooled) (Adapted from Ref.^7^)

Antipsychotic, length of study(ies)	Weight gain ≥ 7% from baseline			Somnolence/sedation adverse events			Akathisia adverse events		
	Rate (%)		NNH(95% CI)	Rate (%)		NNH(95% CI)	Rate (%)		NNH(95% CI)
	Placebo	Drug		Placebo	Drug		Placebo	Drug	
Risperidone/paliperidone formulations									
Risperidone microspheres, 12 wk	6	9	33 (ns)	3^a^	5.4^a^	42 (ns)	6^b^	7.6^b^	69 (ns)
Risperidone subcutaneous (Indivior), 8 wk	18.0	37.6	6 (4–10)	0^a^	7.3^a^	14 (10–26)	4.2	4.7	199 (ns)
Risperidone ISM, 12 wk	4.1	13.7	11 (7–25)	2^a^	3.4^a^	72 (ns)	1.4	5.5	25 (14–108)
Risperidone oral, up to 8 mg/d, up to 8 wk	2.9 ^c^	8.7 ^c^	18 (13–30)	2	10	13 (9–23)	3	10	15 (10–32)
Paliperidone palmitate, 9 and 13 wk	3.3	8.7	19 (13–33)	3^a^	4.7^a^	59 (ns)	3	3.1	1839 (ns)
Paliperidone oral, 3–12 mg/d, up to 6 wk	5	7.9	35 (ns)	7^a^	9.4^a^	42 (ns)	3.9	6.5	40 (ns)
Aripiprazole formulations									
Aripiprazole monohydrate, 12 wk	8.5	21.5	8 (5–21)	1.2	5.4	24 (13–225)	3.5	11.4	13 (8–43)
Aripiprazole lauroxil, 12 wk	5.8	9.2	30 (ns)	1.4	2.2	139 (ns)	4.3	11.6	14 (9–33)
Aripiprazole oral, 2–30 mg/d, up to 6 wk	3.2	8.1	21 (14–42)	8.0	11.0	34 (ns)	6.8	10.0	31 (16–622)
Olanzapine formulations									
Olanzapine pamoate, 8 wk	12.4	28.6	7 (5–13)	7^a^	11.3^a^	24 (ns)	NR	NR	NR
Olanzapine oral, 2.5–20 mg, up to 6 wk	22.2 ^c^	3 ^c^	6 (NC)	15.3	26.2	10 (6–41)	5	1	25 (14–134)

a Pooled term of somnolence/sedation as reported in the product label.

b Pooled term of akathisia/restlessness as reported in the product label.

c Pooled schizophrenia and bipolar as reported in the product label.

CI, confidence interval; NC, the 95% CI is not calculable as denominators were not provided in product labeling; NR, not reported (did not meet threshold for reporting); NNH, number needed to harm; ns, not significant at the *p* < 0.05 threshold and thus the 95% CI is not shown.

## Prevention of relapse with long-acting injectable antipsychotics: what is the evidence?

Although LAI antipsychotics can be used acutely, LAI antipsychotics are more often considered as part of a long-term treatment strategy to minimize the risk of relapse. Real-world prospective and retrospective studies comparing LAI antipsychotics versus oral antipsychotics generally demonstrate decreases in relapse, hospitalization, and all-cause discontinuation for patients receiving LAI antipsychotics,^1^ ^,^^89^ as well as a decrease in all-cause mortality.^90^

Although not without controversy,^91^ placebo-controlled randomized withdrawal study designs are often used to establish efficacy for the maintenance indication. The typical study design would be one where patients with the disease of interest are stabilized on the test medication and then subsequently randomized to either continue the test medication or receive a placebo. The primary outcome measure is usually time to relapse, impending relapse, or recurrence, depending on the disorder and the study. This has been formally assessed versus placebo in registration studies in individuals with schizophrenia for paliperidone palmitate administered monthly^92^ or every 3 months,^93^ olanzapine pamoate,^94^ aripiprazole monohydrate,^95^ and 1-month/2-month risperidone subcutaneous.^96^ Registration studies using similar designs were also done in individuals with bipolar disorder for risperidone microspheres (monotherapy or adjunctive use)^97^ ^,^^98^ and aripiprazole monohydrate (monotherapy),^99^ and in individuals with schizoaffective disorder for once-monthly paliperidone palmitate (monotherapy or adjunctive use).^100^ Number needed to treat versus placebo for prevention of relapse or recurrence for any of the tested medications for any of the indications range from 4 to 8, with overlap of the 95% confidence intervals (Table 8).^29^ ^,^^39^ ^,^^40^ ^,^^49^ ^,^^65^ ^,^^66^ ^,^^96^ ^,^^101^ ^,^^102^ These effect sizes are consistent with the broader literature on maintenance treatment.^103^ In general, there appears to be no clear differences between antipsychotics for relapse prevention, and thus, choice of antipsychotic for maintenance treatment can be guided mainly by tolerability.^104^

**Table 8.** Prevention of Relapse or Recurrence as Quantified Using Number Needed to Treat versus Placebo (or versus 45 mg/4 wk for Olanzapine Pamoate), Data from US Registration Studies of Long-Acting Injectable Antipsychotics (Adapted from Ref.^7^)

Disorder	Antipsychotic	Relapse or recurrence		
		Rate (%)		NNT(95% CI)
		Placebo	Drug	
Schizophrenia	Paliperidone palmitate monthly, flexibly dosed, 39–156 mg/4-wk	34.0	9.6	5 (4–7)
	Paliperidone palmitate 3-mo, flexibly dosed, 273–819 mg/12-wk	29.0	8.8	5 (4–9)
	Risperidone subcutaneous monthly (Teva), dose range 50–125 mg	29.3	7.1	5 (4–7)
	Risperidone subcutaneous 2-mo, dose range 100–250 mg	29.3	12.8	7 (5–13)
	Aripiprazole monohydrate, 400 mg/4-wk	39.6	10.0	4 (3–5)
	Olanzapine pamoate 150 mg/2-wk	29.2	15.7	8 (5–26)
	Olanzapine pamoate 300 mg/2-wk	29.2	5.0	5 (4–7)
	Olanzapine pamoate 405/4-wk	29.2	12.3	6 (4–12)
Schizoaffective disorder	Paliperidone palmitate monthly, flexibly dosed, 78–156 mg/4-wk	33.5	15.2	6 (4–11)
Bipolar disorder	Risperidone microspheres, as adjunctive therapy, flexibly dosed, 25–50 mg/2-wk	45.8	23.1	5 (3–16)
	Risperidone microspheres, as monotherapy, flexibly dosed, 25–50 mg/2-wk	56.3	30.0	4 (3–7)
	Aripiprazole monohydrate, 400 mg/4-wk	51.1	26.5	5 (3–8)

CI, confidence interval; NNT, number needed to treat.

## Selecting a long-acting injectable antipsychotic

The simplest scenario is if the patient is already receiving an antipsychotic that is available as an LAI formulation. Then it is a matter of educating the patient (and caregiver) about the availability of this different way of receiving medication. If there are competing LAI formulations for the same or related molecule, then a review of the “amenities of care” (Table 3) is in order. There may be a preference for a specific injection interval that is available with only some of the products. Patients and caregivers sometimes need to be reassured that a longer interval between injections does not necessarily mean that visits will be scheduled less often. If naïve to the molecule, it will be necessary to administer test doses of the oral formulation in order to rule out potential hypersensitivity as well as assess acute tolerability in general.

There may be some personal preferences regarding subcutaneous versus intramuscular injection on the part of both the patient and the provider.^105^

Principally because of injection volume, not all formulations or doses can be given in the arm and must be administered in the gluteal muscle. Patients who are new to LAI antipsychotics may not be aware that injection is into the upper outer quadrant of the gluteal muscle, and that this location is relatively easy to access without embarrassment. The gauge and length of the needle may be of interest to the patient, who may be reassured when shown the different options available to select from.

In some situations, it may be unrealistic to expect adherence to oral supplementation, and alternatives should be considered. Some choices require an infrastructure (refrigeration for storage of almost all the formulations of risperidone, examination table for administering subcutaneous injections in the abdomen, ability to observe the patient for 3 hours after each injection of olanzapine pamoate). For patients receiving oral fluphenazine or haloperidol and a switch to a LAI antipsychotic is being considered, despite the relatively low cost of haloperidol decanoate or fluphenazine decanoate, the clinician needs to weigh the potential disadvantages of using concomitant oral anticholinergics as discussed earlier. On occasion, the supply chain for older generic medications sometimes gets interrupted^106^; the American Society of Health-System Pharmacists maintains a web resource that tracks drug shortages.^107^

Additional pragmatic concerns for the provider are the presence of drug–drug interactions that cannot be easily remedied because of dosing constraints, as well as the lack of dosing flexibility when considering longer injection intervals. The requirements for reconstitution may be time-consuming and burdensome for some of the agents. Complex re-initiation requirements are present for some formulations; however, in general, the longer the injection interval, the greater the flexibility in the timing of an early or late injection (Table 9).

**Table 9.** Formulations of Long-Acting Injectable Antipsychotics and Recommendations Regarding Early or Late Dosing as per US Product Labels

Formulation (manufacturer)	Early dosing permitted?	Grace period if late (defined as the amount of time that can elapse after an injection is due before any supplemental medication is required)
Risperidone microspheres (Janssen)	No data	None
Risperidone microspheres (Luye)	No data	None
Risperidone subcutaneous (Indivior)	No data	NA^a^
Risperidone subcutaneous (Teva)	No data	NA^a^
Risperidone ISM (Rovi)	No	NA^a^
Paliperidone palmitate 1-mo (Janssen)	21 d after last injection	2 wk
Paliperidone palmitate 3-mo (Janssen)	2.5 mo after last injection	4 wk
Paliperidone palmitate 6-mo (Janssen)	5.5 mo after last injection	3 wk
Paliperidone palmitate 1-mo (Luye)	21 d after last injection	2 wk
Aripiprazole lauroxil (Alkermes)	14 d after last injection	2–4 wk, depending on dose
Aripiprazole monohydrate 1-mo (Otsuka/Lundbeck)	26 d after last injection	1–2 wk, depending on number of injections
Aripiprazole monohydrate 2-mo (Otsuka/Lundbeck)	1½ mo after last injection	6 wk
Olanzapine pamoate (Eli Lilly)	No data	No data

a NA, no oral supplementation or loading dose is required upon initiation with this formulation.

If the patient is receiving acute treatment, available options in the United States that do not require any oral supplementation are paliperidone palmitate, risperidone subcutaneous, and olanzapine pamoate; LAI antipsychotic formulations that can be initiated with the administration of only a single injection are paliperidone palmitate (Luye), risperidone subcutaneous, and olanzapine pamoate. Requiring only 1 dose of oral medication are the 1-day initiation options for aripiprazole monohydrate and aripiprazole lauroxil. Prior knowledge of tolerability and efficacy is important because once injected, the medication cannot be withdrawn. Oral or intramuscular short-acting antipsychotic medications are, in most situations, the most prudent way to initiate antipsychotic treatment in an individual who is treatment-naïve or if a medication history cannot be reliably obtained. A shortened initiation period is especially useful for hospitalized patients being transitioned to outpatient care, and in forensic settings such as jails.

Long-term considerations in maintenance treatment include weight gain and metabolic adverse effects, especially with olanzapine; first-generation LAI antipsychotics could possibly be considered under these circumstances and where a switch among the second-generation LAIs was not helpful. If prolactin-related adverse effects are a clinical concern, one of the aripiprazole LAI formulations would be the first choice; to be avoided under these circumstances would be paliperidone palmitate, risperidone microspheres, or the first-generation LAI antipsychotics.

Cost considerations are sometimes obstacles, and access to patient-assistance programs can be helpful. An important factor is whether reimbursement or enrollment in a patient-assistance program is possible with off-label use of a formulation, even when it would be logical to use it. For example, only risperidone microspheres and aripiprazole monohydrate are approved for maintenance treatment of bipolar I disorder, with risperidone microspheres the only LAI approved in combination with lithium or valproate for this indication, and only paliperidone once monthly is approved for the treatment of schizoaffective disorder. Reimbursement may prevent the use of risperidone subcutaneous, 3- or 6-month paliperidone palmitate, or aripiprazole lauroxil under these circumstances.

## Conclusion

Only 3 second-generation antipsychotics are available as LAI preparations; however, there are multiple options in terms of formulation. Choosing among the different LAI antipsychotics is partly based on pragmatic concerns. For example, olanzapine pamoate would not be a practical option if the mandatory 3-hour post-injection observation period cannot be provided. For patients receiving oral risperidone, using risperidone microspheres can be inconvenient as that formulation is administered every 2 weeks, requires refrigeration and reconstitution, and must be accompanied by oral supplementation after the initial injection. Instead of risperidone microspheres, either 1-month/2-month risperidone subcutaneous or paliperidone palmitate can be considered as these choices do not require oral supplementation upon initiation, entail less frequent injections (either monthly or every other month for 1-month/2-month risperidone subcutaneous, or every 1, 3, or 6 months for paliperidone palmitate), are supplied in prefilled syringes, have a relatively small needle gauge, and is normally stored at room temperature (paliperidone palmitate) or can remain at room temperature for up to 90 days (1-month/2-month risperidone subcutaneous). Regarding aripiprazole LAI, there are 2 competing formulations available in the United States—aripiprazole monohydrate and aripiprazole lauroxil; although both can be initiated in 1 day and administered at the outset every month or every other month, there are differences in terms of dosage strengths and approved indications.

## CNS SPECTRUMS

### Choosing Amongst the Long-Acting Injectable Antipsychotics: An Evidence-Based Pragmatic Guide

This CME activity is provided by HMP Education and Neuroscience Education Institute (NEI).

### CME/CE Information

**Target Audience:** This activity has been developed for the healthcare team or individual prescriber specializing in mental health. All other healthcare team members interested in psychopharmacology are welcome for advanced study.

**Learning Objectives:** After completing this educational activity, you should be better able to:

- Differentiate among the different antipsychotics and the formulations of the same antipsychotic in terms of clinical applications, including treatment of schizophrenia and maintenance therapy in bipolar I disorder

**Accreditation:** In support of improving patient care, this activity has been planned and implemented by HMP Education and Neuroscience Education Institute (NEI). HMP Education is jointly accredited by the Accreditation Council for Continuing Medical Education (ACCME), the Accreditation Council for Pharmacy Education (ACPE), and the American Nurses Credentialing Center (ANCC), to provide continuing education for the healthcare team.

**Activity Overview:** This activity is best supported via a computer or device with current versions of the following browsers: Mozilla Firefox, Google Chrome, or Safari. A PDF reader is required for print publications. A post-test score of 70% or higher is required to receive CME/CE credit.

**Estimated Time to Complete:** 1 hour.

Continuing Education credit will be available for three (3) years from the publication date of the associated article. Please visit https://nei.global/cnsspectrums2025 for additional information and to access the CE activity.

**NEI maintains a record of participation for six (6) years.*

**Instructions for Optional Posttest and CME Credit**

1. Read the article
2. Successfully complete the posttest at https://nei.global/CNS/LAI-04
3. Print your certificate

Questions? Email customerservice@neiglobal.com.

**Credit Designations:** The following are being offered for this activity:

- **Physician: ACCME *AMA PRA Category 1 Credits*™.**
  - HMP Education designates this enduring material for a maximum of 1.00 *AMA PRA Category 1 Credit*™. Physicians should claim only the credit commensurate with the extent of their participation in the activity.
- **Nurse: ANCC contact hours.**
  - This continuing nursing education activity awards 1.00 contact hour. Provider approved by the California Board of Registered Nursing, Provider #18006 for 1.00 contact hour.
- **Nurse Practitioner: ACCME *AMA PRA Category 1 Credit*™.**
  - American Academy of Nurse Practitioners National Certification Program accepts *AMA PRA Category 1 Credits*™ from organizations accredited by the ACCME.
  - The content in this activity pertaining to pharmacology is worth 1.00 continuing education hour of pharmacotherapeutics.
- **Pharmacy: ACPE application-based contact hours.**
  - This internet enduring, knowledge-based activity has been approved for a maximum of 1.00 contact hour (.10 CEU).
  - *The official record of credit will be in the CPE Monitor system. Following ACPE Policy, NEI and HMP Education must transmit your claim to CPE Monitor within 60 days from the date you complete this CPE activity and are unable to report your claimed credit after this 60-day period. Ensure your profile includes your DOB and NABP ID.*
- **Physician Associate/Assistant: AAPA Category 1 CME credits.**
  **○**
  - HMP Education has been authorized by the American Academy of PAs (AAPA) to award AAPA Category 1 CME credits for activities planned in accordance with the AAPA CME Criteria. This internet enduring activity is designated for 1.00 AAPA Category 1 credit. Approval is valid until February 11, 2028. PAs should only claim credit commensurate with the extent of their participation.
- **Psychology: APA CE credits.**
  **○**
  - Continuing Education (CE) credits for psychologists are provided through the co-sponsorship of the American Psychological Association (APA) Office of Continuing Education in Psychology (CEP). The APA CEP Office maintains responsibility for the content of the programs. This activity awards 1.00 CE Credit.
- **Social Work: ASWB-ACE CE credits.**
  **○**
  - As a Jointly Accredited Organization, HMP Education is approved to offer social work continuing education by the Association of Social Work Boards (ASWB) Approved Continuing Education (ACE) program. Organizations, not individual courses, are approved under this program. Regulatory boards are the final authority on courses accepted for continuing education credit. Social workers completing this internet enduring course receive general continuing education credit.
- **Non-Physician Member of the Healthcare Team: Certificate of Participation.**
  **○**
  - HMP Education awards hours of participation (consistent with the designated number of *AMA PRA Category 1 Credit*™) to a participant who successfully completes this educational activity.

**Peer Review:** The content was peer-reviewed by an MD, MPH specializing in forensics, psychosis, schizophrenia, mood disorders, anxiety, and cognitive disorders — to ensure the scientific accuracy and medical relevance of information presented and its independence from commercial bias. NEI and HMP Education take responsibility for the content, quality, and scientific integrity of this CME/CE activity.

**Disclosures:** All individuals in a position to influence or control content are required to disclose any relevant financial relationships. Any relevant financial relationships were mitigated prior to the activity being planned, developed, or presented.
***Faculty Author / Presenter***

**Leslie Citrome, MD, MPH**
*Clinical Professor of Psychiatry and Behavioral Sciences, New York Medical College, Valhalla, NY* Consultant/Advisor: AbbVie/Allergan, Acadia, Adamas, Alkermes, Angelini, Astellas, Avanir, Axsome, Biogen, BioXcel, Bristol-Myers Squibb, Boehringer Ingelheim, Cadent Therapeutics, Cerevel, Clinilabs, COMPASS, Delpor, Eisai, Enteris BioPharma, HLS Therapeutics, Idorsia, INmune Bio, Impel, Intra-Cellular Therapies, Janssen, Karuna, Lundbeck, Luye, Lyndra, MapLight, Marvin, Medavante-ProPhase, Merck, Mitsubishi-Tanabe Pharma, Neumora, Neurocrine, Neurelis, Noema, Novartis, Noven, Otsuka, Ovid, Praxis, Recordati, Relmada, Reviva, Sage, Sumitomo/Sunovion, Supernus, Teva, University of Arizona, Vanda, Wells Fargo, and ad hoc consulting for marketing, commercial, or scientific scoping research Speakers Bureau: AbbVie/Allergan, Acadia, Alkermes, Angelini, Axsome, BioXcel, Bristol-Myers Squibb, Eisai, Idorsia, Intra-Cellular Therapies, Janssen, Lundbeck, Neurocrine,f Noven, Otsuka, Recordati, Sage, Sunovion, Takeda, Teva, Vanda, and CME activities organized by Medscape, NACCME, NEI, Vindico, and Universities/Professional Organizations Stockholder: AbbVie/Allergan, Acadia, Alkermes, Angelini, Axsome, BioXcel, Bristol-Myers Squibb, Eisai, Idorsia, Intra-Cellular Therapies, Janssen, Lundbeck, Neurocrine,f Noven, Otsuka, Recordati, Sage, Sunovion, Takeda, Teva, Vanda, and CME activities organized by Medscape, NACCME, NEI, Vindico, and Universities/Professional Organizations Stock Options: Reviva Royalties/Publishing: Taylor & Francis (Editor-in-Chief, Current Medical Research and Opinion), Wiley (Editor-in-Chief, International Journal of Clinical Practice, through 2019), UpToDate (Reviewer), Springer Healthcare (Book), Elsevier (Topic Editor, Psychiatry, Clinical Therapeutics) The remaining Planning Committee members, Content Editors, Peer Reviewer, and NEI planners/staff have no financial relationships to disclose. NEI and HMP Education planners and staff include Meghan Grady, Caroline O’Brien, MS, Ali Holladay, Moriah Carswell, Andrea Zimmerman, EdD, CHCP, Brielle Calleo, and Steven S. Simring, MD, MPH.
**Disclosure of Off-Label Use:** This educational activity may include discussion of unlabeled and/or investigational uses of agents that are not currently labeled for such use by the FDA. Please consult the product prescribing information for full disclosure of labeled uses.
**Cultural Linguistic Competency and Implicit Bias:** A variety of resources addressing cultural and linguistic competencies and strategies for understanding and reducing implicit bias can be found in this handout—download me.

## Accessibility Statement

For questions regarding this educational activity, or to cancel your account, please email customerservice@neiglobal.com.

**Support:** This activity is supported by an unrestricted educational grant from Alkermes, Inc., Teva Pharmaceuticals, Johnson & Johnson Innovative Medicine and Otsuka America Pharmaceutical, Inc.

## References

[1] Kishimoto T, Hagi K, Kurokawa S, et al. Long-acting injectable versus oral antipsychotics for the maintenance treatment of schizophrenia: a systematic review and comparative meta-analysis of randomised, cohort, and pre-post studies. Lancet Psychiatry. 2021;8(5):387–404. 33862018 10.1016/S2215-0366(21)00039-0

[2] Correll CU, Solmi M, Croatto G, et al. Mortality in people with schizophrenia: a systematic review and meta-analysis of relative risk and aggravating or attenuating factors. World Psychiatry. 2022;21(2):248–271. 35524619 10.1002/wps.20994 PMC9077617

[3] Keepers GA, Fochtmann LJ, Anzia JM, et al. The American Psychiatric Association practice guideline for the treatment of patients with schizophrenia. Am J Psychiatry. 2020;177(9):868–872. 32867516 10.1176/appi.ajp.2020.177901

[4] Subotnik KL, Casaus LR, Ventura J, et al. Long-acting injectable risperidone for relapse prevention and control of breakthrough symptoms after a recent first episode of schizophrenia. a randomized clinical trial. JAMA Psychiatry. 2015;72(8):822–829. 26107752 10.1001/jamapsychiatry.2015.0270 PMC5065351

[5] Kane JM, Schooler NR, Marcy P, et al. Effect of long-acting injectable antipsychotics vs usual care on time to first hospitalization in early-phase schizophrenia: a randomized clinical trial. JAMA Psychiatry. 2020;77(12):1217–1224. Erratum in: *JAMA Psychiatry.* 2020;77(12):1310. 32667636 10.1001/jamapsychiatry.2020.2076 PMC7364341

[6] Tiihonen J, Haukka J, Taylor M, et al. A nationwide cohort study of oral and depot antipsychotics after first hospitalization for schizophrenia. Am J Psychiatry. 2011;168(6):603–609. Erratum in *Am J Psychiatry.* 2012;169(2):223. 21362741 10.1176/appi.ajp.2011.10081224

[7] Citrome L. Long-acting injectable antipsychotics: what, when, and how. CNS Spectr. 2021;26(2):118–129. 10.1017/S1092852921000456 33928884

[8] Vanegas-Arroyave N, Caroff SN, Citrome L, et al. An evidence-based update on anticholinergic use for drug-induced movement disorders. CNS Drugs. 2024;38(4):239–254. 38502289 10.1007/s40263-024-01078-z PMC10980662

[9] Mancini V, Latreche C, Fanshawe JB, et al. Anticholinergic burden and cognitive function in psychosis: a systematic review and meta-analysis. Am J Psychiatry 2025; 182 (4):349–359. 40007252 10.1176/appi.ajp.20240260

[10] Vinogradov S, Fisher M, Warm H, et al. The cognitive cost of anticholinergic burden: decreased response to cognitive training in schizophrenia. Am J Psychiatry. 2009;166(9):1055–1062. 19570929 10.1176/appi.ajp.2009.09010017 PMC3735363

[11] Carbon M, Kane JM, Leucht S, et al. Tardive dyskinesia risk with first- and second-generation antipsychotics in comparative randomized controlled trials: a meta-analysis. World Psychiatry. 2018;17(3):330–340. 30192088 10.1002/wps.20579 PMC6127753

[12] Carbon M, Hsieh CH, Kane JM, et al. Tardive dyskinesia prevalence in the period of second-generation antipsychotic use: a meta-analysis. J Clin Psychiatry. 2017;78(3):e264–e278. 28146614 10.4088/JCP.16r10832

[13] Taylor D. Psychopharmacology and adverse effects of antipsychotic long-acting injections: a review. Br J Psychiatry Suppl. 2009;52:S13–19. 19880912 10.1192/bjp.195.52.s13

[14] Meftah AM, Deckler E, Citrome L, et al. New discoveries for an old drug: a review of recent olanzapine research. Postgrad Med. 2020;132(1):80–90. 31813311 10.1080/00325481.2019.1701823

[15] Citrome L, McEvoy JP, Todtenkopf MS, et al. A commentary on the efficacy of olanzapine for the treatment of schizophrenia: the past, present, and future. Neuropsychiatr Dis Treat. 2019;15:2559–2569. 31564881 10.2147/NDT.S209284 PMC6733343

[16] Citrome L. Paliperidone: quo vadis? Int J Clin Pract. 2007;61(4):653–662. 17343660 10.1111/j.1742-1241.2007.01321.x

[17] Huhn M, Nikolakopoulou A, Schneider-Thoma J, et al. Comparative efficacy and tolerability of 32 oral antipsychotics for the acute treatment of adults with multi-episode schizophrenia: a systematic review and network meta-analysis. Lancet. 2019;394(10202):939–951. Erratum in *Lancet.* 2019;394(10202):918. 31303314 10.1016/S0140-6736(19)31135-3 PMC6891890

[18] Faden J, Citrome L. Resistance is not futile: treatment-refractory schizophrenia: overview, evaluation and treatment. Expert Opin Pharmacother. 2019;20(1):11–24. 30407873 10.1080/14656566.2018.1543409

[19] Howes OD, McCutcheon R, Agid O, et al. Treatment-resistant schizophrenia: treatment response and resistance in psychosis (TRRIP) working group consensus guidelines on diagnosis and terminology. Am J Psychiatry. 2017;174(3):216–229. 27919182 10.1176/appi.ajp.2016.16050503 PMC6231547

[20] Volavka J, Citrome L. Oral antipsychotics for the treatment of schizophrenia: heterogeneity in efficacy and tolerability should drive decision-making. Expert Opin Pharmacother. 2009;10(12):1917–1928. 19558339 10.1517/14656560903061309

[21] Citrome L, Ketter TA. When does a difference make a difference? Interpretation of number needed to treat, number needed to harm, and likelihood to be helped or harmed. Int J Clin Pract. 2013;67(5):407–411. 23574101 10.1111/ijcp.12142

[22] Citrome L, Neugebauer NM, Meli AA, Kando J. Xanomeline and trospium chloride versus placebo for the treatment of schizophrenia: a post hoc analysis of number needed to treat, number needed to harm, and likelihood to be helped or harmed. Neuropsychiatr Dis Treat. 2025;21:761–773. 40212458 10.2147/NDT.S503494 PMC11981872

[23] Citrome L. New second-generation long-acting injectable antipsychotics for the treatment of schizophrenia. Expert Rev Neurother. 2013;13(7):767–783. 23898849 10.1586/14737175.2013.811984

[24] Eugia US. Fluphenazine decanoate: fluphenazine decanoate injection, solution. Prescribing information. Revised November 2024. Accessed April 14, 2025. https://dailymed.nlm.nih.gov/dailymed/getFile.cfm?setid=1f532edc-1a2c-4221-80dd-1ac158dd3a72&type=pdf.

[25] Janssen. HALDOL Decanoate 50 (haloperidol), HALDOL Decanoate 100 (haloperidol), For IM Injection Only. Prescribing Information. Revised January 2025. Accessed April 14, 2025. http://www.janssenlabels.com/package-insert/product-monograph/prescribing-information/HALDOL+Decanoate-pi.pdf.

[26] Correll CU, Ahn T, Bar-Nur A, et al. The SOLARIS protocol: a phase 3, randomized, double-blind, placebo-controlled trial assessing safety and efficacy of TV-44749 in adults with schizophrenia. Neurosci Appl. 2023; 2(Supp l2):103589.

[27] Ehret MJ, Fuller MA. Long-acting injectable risperidone. Ann Pharmacother. 2004;38(12):2122–7. Erratum in *Ann Pharmacother.* 2005;39(1):201. 15522979 10.1345/aph.1E085

[28] Harrison TS, Goa KL. Long-acting risperidone: a review of its use in schizophrenia. CNS Drugs. 2004;18(2):113–132. 14728058 10.2165/00023210-200418020-00005

[29] Citrome L. Long-acting injectable antipsychotics update: lengthening the dosing interval and expanding the diagnostic indications. Expert Rev Neurother. 2017;17(10):1029–1043. 28832262 10.1080/14737175.2017.1371014

[30] Janssen. RISPERDAL CONSTA (risperidone) long-acting injection. Prescribing information. Revised January 2025. Accessed April 14, 2025. http://www.janssenlabels.com/package-insert/product-monograph/prescribing-information/RISPERDAL+CONSTA-pi.pdf.

[31] Eerdekens M, Van Hove I, Remmerie B, et al. Pharmacokinetics and tolerability of long-acting risperidone in schizophrenia. Schizophr Res. 2004;70(1):91–100. 15246468 10.1016/j.schres.2003.11.001

[32] Luye. RYKINDO (risperidone) for extended-release injectable suspension, for intramuscular use. Prescribing Information. Revised May 2023. Accessed April 14, 2025. https://www.luye.cn/lvye_en/rykindo.pdf.

[33] Faden J, Ramirez C, Martinez V, et al. An overview of the currently available and emerging long-acting formulations of risperidone for schizophrenia and bipolar disorder. Expert Rev Neurother. 2024;24(8):761–771. 39044342 10.1080/14737175.2024.2370349

[34] Indivior. PERSERIS (risperidone) for extended-release injectable suspension, for subcutaneous use. Prescribing Information. Revised January 2025. Accessed April 14, 2025. https://www.perserishcp.com/prescribing-information.pdf.

[35] Indivior. Indivior provides business update. July 9, 2024. Accessed April 14, 2025. https://otp.tools.investis.com/clients/uk/indivior2/rns/regulatory-story.aspx?newsid=1840448.

[36] Teva. UZEDY (risperidone) extended-release injectable suspension, for subcutaneous use. Prescribing Information. Revised January 2025. Accessed April 14, 2025. https://www.uzedy.com/globalassets/uzedy/prescribing-information.pdf.

[37] Rovi. RISVAN (risperidone) for extended-release injectable suspension, for intramuscular use. Prescribing Information. Revised March 2024. Accessed April 14, 2025. https://www.accessdata.fda.gov/drugsatfda_docs/label/2024/214835s000lbl.pdf.

[38] Janssen. INVEGA SUSTENNA (paliperidone palmitate) extended-release injectable suspension, for intramuscular use. Prescribing Information. Revised January 2025. Accessed April 14, 2025. http://www.janssenlabels.com/package-insert/product-monograph/prescribing-information/INVEGA+SUSTENNA-pi.pdf.

[39] Citrome L. Paliperidone palmitate: review of the efficacy, safety and cost of a new second-generation depot antipsychotic medication. Int J Clin Pract. 2010;64(2):216–239. 19886879 10.1111/j.1742-1241.2009.02240.x

[40] Greenberg WM, Citrome L. Paliperidone palmitate for schizoaffective disorder: a review of the clinical evidence. Neurol Ther. 2015;4(2):81–91. 26662360 10.1007/s40120-015-0030-4 PMC4685865

[41] Russu A, Kern Sliwa J, Ravenstijn P, et al. Maintenance dose conversion between oral risperidone and paliperidone palmitate 1 month: Practical guidance based on pharmacokinetic simulations. Int J Clin Pract. 2018;72(6):e13089. 29707876 10.1111/ijcp.13089 PMC6175146

[42] Chue P, Chue J. A critical appraisal of paliperidone long-acting injection in the treatment of schizoaffective disorder. Ther Clin Risk Manag. 2016;12:109–116. 26869795 10.2147/TCRM.S81581 PMC4737499

[43] Luye. ERZOFRI (paliperidone palmitate) extended-release injectable suspension, for intramuscular use. Prescribing Information. Revised January 2025. Accessed April 14, 2025. https://www.accessdata.fda.gov/drugsatfda_docs/label/2025/216352s001lbl.pdf.

[44] Faden J, Citrome L. A new paliperidone palmitate formulation: how is it different and where does it fit in our array of choices for long-acting formulations of risperidone and paliperidone? Curr Med Res Opin. 2025; 41(4):663–666. 40126382 10.1080/03007995.2025.2482654

[45] Janssen. INVEGA TRINZA (paliperidone palmitate) extended-release injectable suspension, for intramuscular use. Prescribing Information. Revised January 2025. Accessed April 14, 2025. http://www.janssenlabels.com/package-insert/product-monograph/prescribing-information/INVEGA+TRINZA-pi.pdf.

[46] Ravenstijn P, Remmerie B, Savitz A, et al. Pharmacokinetics, safety, and tolerability of paliperidone palmitate 3-month formulation in patients with schizophrenia: a phase-1, single-dose, randomized, open-label study. J Clin Pharmacol. 2016;56(3):330–339. 26189570 10.1002/jcph.597

[47] Janssen. INVEGA HAFYERA (paliperidone palmitate) extended-release injectable suspension, for gluteal intramuscular use. Prescribing Information. Revised January 2025. Accessed April 14, 2025. https://www.janssenlabels.com/package-insert/product-monograph/prescribing-information/INVEGA+HAFYERA-pi.pdf.

[48] Faden J, Citrome L. How would you like to take your medicine 2 times a year? Paliperidone palmitate every 6 months for the maintenance treatment of schizophrenia. Clin Ther. 2022;44(4):476–479. 35369994 10.1016/j.clinthera.2022.02.003

[49] Citrome L. Aripiprazole long-acting injectable formulations for schizophrenia: aripiprazole monohydrate and aripiprazole lauroxil. Expert Rev Clin Pharmacol. 2016;9(2):169–186. 26573020 10.1586/17512433.2016.1121809

[50] Ehret MJ, Davis E, Luttrell SE, et al. Aripiprazole lauroxil NanoCrystal Dispersion technology (Aristada initio). Clin Schizophr Relat Psychoses. 2018;12(2):92–96. 30040476

[51] Hard ML, Wehr A, von Moltke L, et al. Pharmacokinetics and safety of deltoid or gluteal injection of aripiprazole lauroxil NanoCrystal Dispersion used for initiation of the long-acting antipsychotic aripiprazole lauroxil. Ther Adv Psychopharmacol. 2019;9:2045125319859964. 31308935 10.1177/2045125319859964 PMC6607563

[52] Otsuka. ABILIFY MAINTENA (aripiprazole) for extended-release injectable suspension, for intramuscular use. Prescribing information. Revised March 2025. Accessed April 14, 2025. https://www.otsuka-us.com/sites/g/files/qhldwo2966/files/media/static/Abilify-M-PI.pdf.

[53] Otsuka. ABILIFY ASIMTUFII® (aripiprazole) extended-release injectable suspension, for intramuscular use. Prescribing information. Revised March 2025. Accessed April 14, 2025. https://www.otsuka-us.com/media/static/Abilify-Asimtufii-PI.pdf.

[54] Alkermes. ARISTADA (aripiprazole lauroxil) extended-release injectable suspension, for intramuscular use. Prescribing Information. Revised January 2025. Accessed April 14, 2025. https://www.aristadahcp.com/downloadables/ARISTADA-PI.pdf.

[55] Alkermes. ARISTADA INITIO (aripiprazole lauroxil) extended-release injectable suspension, for intramuscular use. Prescribing information. Revised January 2025. Accessed April 14, 2025. https://www.aristadahcp.com/downloadables/ARISTADA-INITIO-PI.pdf.

[56] Hard ML, Wehr AY, Du Y, et al. Pharmacokinetic evaluation of a 1-day treatment initiation option for starting long-acting aripiprazole lauroxil for schizophrenia. J Clin Psychopharmacol. 2018;38(5):435–441. 30015676 10.1097/JCP.0000000000000921 PMC6133194

[57] Raoufinia A, Peters-Strickland T, Nylander AG, et al. Aripiprazole once-monthly 400 mg: comparison of pharmacokinetics, tolerability, and safety of deltoid versus gluteal administration. Int J Neuropsychopharmacol. 2017;20(4):295–304. 28204607 10.1093/ijnp/pyw116 PMC5409034

[58] Fagiolini A, Leopold K, Pappa S, et al. Survey on the initiation of aripiprazole once-monthly via a two-injection start in adult patients with schizophrenia: experience of european healthcare professionals. Adv Ther. 2025;42(4):1935–1949. 40025389 10.1007/s12325-025-03130-w PMC11929703

[59] Harlin M, Yildirim M, Such P, et al. A randomized, open-label, multiple-dose, parallel-arm, pivotal study to evaluate the safety, tolerability, and pharmacokinetics of aripiprazole 2-month long-acting injectable in adults with schizophrenia or bipolar I disorder. CNS Drugs. 2023;37(4):337–350. 36961650 10.1007/s40263-023-00996-8 PMC10126081

[60] McIntyre RS, Such P, Yildirim M, et al. Safety and efficacy of aripiprazole 2-month ready-to-use 960 mg: secondary analysis of outcomes in adult patients with bipolar I disorder in a randomized, open-label, parallel-arm, pivotal study. Curr Med Res Opin. 2023;39(7):1021–1030. 37272079 10.1080/03007995.2023.2219155

[61] Citrome L, Such P, Yildirim M, et al. Safety and efficacy of aripiprazole 2-month ready-to-use 960 mg: secondary analysis of outcomes in adult patients with schizophrenia in a randomized, open-label, parallel-arm, pivotal study. J Clin Psychiatry. 2023;84(5):23m14873. 10.4088/JCP.23m14873 37672016

[62] Citrome L, Correll CU, Cutler AJ, et al. Aripiprazole lauroxil: development and evidence-based review of a long-acting injectable atypical antipsychotic for the treatment of schizophrenia. Neuropsychiatr Dis Treat. 2025;21:575–596. 40110113 10.2147/NDT.S499367 PMC11921517

[63] Sommi RW, Rege B, Wehr A, et al. Aripiprazole lauroxil dosing regimens: understanding dosage strengths and injection intervals. CNS Spectr. 2022;27(3):262–267. 33267924 10.1017/S1092852920002072

[64] Eli Lilly. ZYPREXA RELPREVV (olanzapine) for extended release injectable suspension. Prescribing information. Revised February 2021. Accessed April 14, 2025. http://pi.lilly.com/us/zyprexa_relprevv.pdf.

[65] Citrome L. Olanzapine pamoate: a stick in time? A review of the efficacy and safety profile of a new depot formulation of a second-generation antipsychotic. Int J Clin Pract. 2009;63(1):140–150. 18834452 10.1111/j.1742-1241.2008.01900.x

[66] Citrome L. Patient perspectives in the development and use of long-acting antipsychotics in schizophrenia: focus on olanzapine long-acting injection. Patient Prefer Adherence. 2009;3:345–355. 20016798 10.2147/ppa.s5734 PMC2792872

[67] Krtalic I, Juretic M, Komlosi A, et al. A long-acting subcutaneous injectable formulation of olanzapine is designed to eliminate the causes of post-injection delirium/sedation syndrome. Neurosci Appl. 2024;3(Suppl 2):104320.

[68] Detke HC, McDonnell DP, Brunner E, et al. Post-injection delirium/sedation syndrome in patients with schizophrenia treated with olanzapine long-acting injection, I: analysis of cases. BMC Psychiatry. 2010;10:43. 20537128 10.1186/1471-244X-10-43 PMC2895589

[69] Luedecke D, Schöttle D, Karow A, et al. Post-injection delirium/sedation syndrome in patients treated with olanzapine pamoate: mechanism, incidence, and management. CNS Drugs. 2015;29(1):41–46. 25424243 10.1007/s40263-014-0216-9

[70] Pandina GJ, Lindenmayer JP, Lull J, et al. A randomized, placebo-controlled study to assess the efficacy and safety of 3 doses of paliperidone palmitate in adults with acutely exacerbated schizophrenia. J Clin Psychopharmacol. 2010;30(3):235–244. Erratum in *J Clin Psychopharmacol.* 2010;30(4):364. 20473057 10.1097/JCP.0b013e3181dd3103

[71] Gopal S, Hough DW, Xu H, et al. Efficacy and safety of paliperidone palmitate in adult patients with acutely symptomatic schizophrenia: a randomized, double-blind, placebo-controlled, dose-response study. Int Clin Psychopharmacol. 2010;25(5):247–256. 20389255 10.1097/YIC.0b013e32833948fa

[72] Nasrallah HA, Gopal S, Gassmann-Mayer C, et al. A controlled, evidence-based trial of paliperidone palmitate, a long-acting injectable antipsychotic, in schizophrenia. Neuropsychopharmacology. 2010;35(10):2072–2082. 20555312 10.1038/npp.2010.79 PMC3055301

[73] Kramer M, Litman R, Hough D, et al. Paliperidone palmitate, a potential long-acting treatment for patients with schizophrenia. Results of a randomized, double-blind, placebo-controlled efficacy and safety study. Int J Neuropsychopharmacol. 2010;13(5):635–647. 19941696 10.1017/S1461145709990988

[74] Lauriello J, Lambert T, Andersen S, et al. An 8-week, double-blind, randomized, placebo-controlled study of olanzapine long-acting injection in acutely ill patients with schizophrenia. J Clin Psychiatry. 2008;69(5):790–799. Erratum in *J Clin Psychiatry.* 2011;72(8):1157. 18452346 10.4088/jcp.v69n0512

[75] Kane JM, Peters-Strickland T, Baker RA, et al. Aripiprazole once-monthly in the acute treatment of schizophrenia: findings from a 12-week, randomized, double-blind, placebo-controlled study. J Clin Psychiatry. 2014;75(11):1254–1260. 25188501 10.4088/JCP.14m09168

[76] Meltzer HY, Risinger R, Nasrallah HA, et al. A randomized, double-blind, placebo-controlled trial of aripiprazole lauroxil in acute exacerbation of schizophrenia. J Clin Psychiatry. 2015;76(8):1085–1090. 26114240 10.4088/JCP.14m09741

[77] Nasser AF, Henderson DC, Fava M, et al. Efficacy, safety, and tolerability of RBP-7000 once-monthly risperidone for the treatment of acute schizophrenia: an 8-week, randomized, double-blind, placebo-controlled, multicenter phase 3 study. J Clin Psychopharmacol. 2016;36(2):130–140. 26862829 10.1097/JCP.0000000000000479

[78] Correll CU, Litman RE, Filts Y, et al. Efficacy and safety of once-monthly risperidone ISM in schizophrenic patients with an acute exacerbation. NPJ Schizophr. 2020;6(1):37. 33239746 10.1038/s41537-020-00127-y PMC7688968

[79] Vita G, Pollini D, Canozzi A, et al. Efficacy and acceptability of long-acting antipsychotics in acutely ill individuals with schizophrenia-spectrum disorders: a systematic review and network meta-analysis. Psychiatry Res. 2024;340:116124. 39173348 10.1016/j.psychres.2024.116124

[80] Citrome L, Du Y, Risinger R, Stankovic S, et al. Effect of aripiprazole lauroxil on agitation and hostility in patients with schizophrenia. Int Clin Psychopharmacol. 2016;31(2):69–75. 26517202 10.1097/YIC.0000000000000106

[81] Citrome L, Volavka J. Specific anti-hostility effects of atypical antipsychotics in persons with schizophrenia: from clozapine to cariprazine. Harv Rev Psychiatry. 2021;29(1):20–34. 33417374 10.1097/HRP.0000000000000275

[82] Citrome L. Sustained-release risperidone via subcutaneous injection: a systematic review of RBP-7000 (PERSERIS) for the treatment of schizophrenia. Clin Schizophr Relat Psychoses. 2018;12(3):130–141. 30339052

[83] Citrome L. Activating and sedating adverse effects of second-generation antipsychotics in the treatment of schizophrenia and major depressive disorder: absolute risk increase and number needed to harm. J Clin Psychopharmacol. 2017;37(2):138–147. 28141623 10.1097/JCP.0000000000000665

[84] Janssen. RISPERDAL (risperidone) tablets, for oral use; RISPERDAL (risperidone) oral solution; RISPERDAL M-TAB (risperidone) orally disintegrating tablets. Prescribing information. Revised January 2025. Accessed April 14, 2025. http://www.janssenlabels.com/package-insert/product-monograph/prescribing-information/RISPERDAL-pi.pdf.

[85] Janssen. INVEGA (paliperidone) extended-release tablets. Prescribing information. Revised January 2025. Accessed April 14, 2025. https://www.janssenlabels.com/package-insert/product-monograph/prescribing-information/INVEGA-pi.pdf.

[86] Otsuka. ABILIFY (aripiprazole) tablets, for oral use. Prescribing information. Revised January 2025. Accessed April 14, 2025. https://www.otsuka-us.com/media/static/Abilify-PI.pdf.

[87] Lilly. ZYPREXA (olanzapine) tablet for oral use; ZYPREXA ZYDIS (olanzapine) tablet, orally disintegrating for oral use; ZYPREXA IntraMuscular (olanzapine) injection, powder, for solution for intramuscular use. Prescribing information. Revised February 2021. Accessed April 14, 2025. https://pi.lilly.com/us/zyprexa-pi.pdf.

[88] Wang D, Schneider-Thoma J, Siafis S, et al. Efficacy, acceptability and side-effects of oral versus long-acting- injectables antipsychotics: systematic review and network meta-analysis. Eur Neuropsychopharmacol. 2024;83:11–18. 38490016 10.1016/j.euroneuro.2024.03.003

[89] Kirson NY, Weiden PJ, Yermakov S, et al. Efficacy and effectiveness of depot versus oral antipsychotics in schizophrenia: synthesizing results across different research designs. J Clin Psychiatry. 2013;74(6):568–575. 23842008 10.4088/JCP.12r08167

[90] Aymerich C, Salazar de Pablo G, Pacho M, et al. All-cause mortality risk in long-acting injectable versus oral antipsychotics in schizophrenia: a systematic review and meta-analysis. Mol Psychiatry. 2025;30(1):263–271. 39174648 10.1038/s41380-024-02694-3 PMC11649555

[91] Emsley R, Fleischhacker WW, Galderisi S, et al. Placebo controls in clinical trials: concerns about use in relapse prevention studies in schizophrenia. BMJ. 2016;354:i4728. 27613560 10.1136/bmj.i4728

[92] Hough D, Gopal S, Vijapurkar U, et al. Paliperidone palmitate maintenance treatment in delaying the time-to-relapse in patients with schizophrenia: a randomized, double-blind, placebo-controlled study. Schizophr Res. 2010;116(2–3):107–117. 19959339 10.1016/j.schres.2009.10.026

[93] Berwaerts J, Liu Y, Gopal S, et al. Efficacy and safety of the 3-month formulation of paliperidone palmitate vs placebo for relapse prevention of schizophrenia: a randomized clinical trial. JAMA Psychiatry. 2015;72(8):830–839. 25820612 10.1001/jamapsychiatry.2015.0241

[94] Kane JM, Detke HC, Naber D, et al. Olanzapine long-acting injection: a 24-week, randomized, double-blind trial of maintenance treatment in patients with schizophrenia. Am J Psychiatry. 2010;167(2):181–189. 20008947 10.1176/appi.ajp.2009.07081221

[95] Kane JM, Sanchez R, Perry PP, et al. Aripiprazole intramuscular depot as maintenance treatment in patients with schizophrenia: a 52-week, multicenter, randomized, double-blind, placebo-controlled study. J Clin Psychiatry. 2012;73(5):617–624. 22697189 10.4088/JCP.11m07530

[96] Kane JM, Harary E, Eshet R, et al. Efficacy and safety of TV-46000, a long-acting, subcutaneous, injectable formulation of risperidone, for schizophrenia: a randomised clinical trial in the USA and Bulgaria. Lancet Psychiatry. 2023;10(12):934–943. 37924833 10.1016/S2215-0366(23)00288-2

[97] Macfadden W, Alphs L, Haskins JT, et al. A randomized, double-blind, placebo-controlled study of maintenance treatment with adjunctive risperidone long-acting therapy in patients with bipolar I disorder who relapse frequently. Bipolar Disord. 2009;11(8):827–839. 19922552 10.1111/j.1399-5618.2009.00761.x

[98] Quiroz JA, Yatham LN, Palumbo JM, et al. Risperidone long-acting injectable monotherapy in the maintenance treatment of bipolar I disorder. Biol Psychiatry. 2010;68(2):156–162. 20227682 10.1016/j.biopsych.2010.01.015

[99] Calabrese JR, Sanchez R, Jin N, et al. Efficacy and safety of aripiprazole once-monthly in the maintenance treatment of bipolar I disorder: a double-blind, placebo-controlled, 52-week randomized withdrawal study. J Clin Psychiatry. 2017;78(3):324–331. 28146613 10.4088/JCP.16m11201

[100] Fu DJ, Turkoz I, Simonson RB, et al. Paliperidone palmitate once-monthly reduces risk of relapse of psychotic, depressive, and manic symptoms and maintains functioning in a double-blind, randomized study of schizoaffective disorder. J Clin Psychiatry. 2015;76(3):253–262. 25562685 10.4088/JCP.14m09416

[101] US Food and Drug Administration. Drug approval package, Zyprexa Relprevv (olanzapine) for extended release injectable suspension 210 mg, 300 mg, and 405 mg, Company: Eli Lilly and Company, Application No.: 022173, Approval Date: 12/11/2009 Accessed April 14, 2025. https://www.accessdata.fda.gov/drugsatfda_docs/nda/2009/022173_zyprexa_relprevv_toc.cfm.

[102] Citrome L, Tohami O, Sharon N, et al. Clinical Benefit and risk profile of TV-46000 for patients with schizophrenia as assessed by number needed to treat (NNT) and number needed to harm (NNH). Poster Session III, S86. 2024 Annual Congress of the Schizophrenia International Research Society, Florence, Italy, April 3–7, 2024.

[103] Ceraso A, Lin JJ, Schneider-Thoma J, et al. Maintenance treatment with antipsychotic drugs for schizophrenia. Cochrane Database Syst Rev. 2020;8:CD008016. 32840872 10.1002/14651858.CD008016.pub3 PMC9702459

[104] Schneider-Thoma J, Chalkou K, Dörries C, et al. Comparative efficacy and tolerability of 32 oral and long-acting injectable antipsychotics for the maintenance treatment of adults with schizophrenia: a systematic review and network meta-analysis. Lancet. 2022;399(10327):824–836. 35219395 10.1016/S0140-6736(21)01997-8

[105] Robinson DG, Suett M, Wilhelm A, et al. Patient and healthcare professional preferences for characteristics of long-acting injectable antipsychotic agents for the treatment of schizophrenia. Adv Ther. 2023;40(5):2249–2264. 36905498 10.1007/s12325-023-02455-8 PMC10129959

[106] Demers MF, Bilodeau I, Laberge L, et al. SU112. The dark side of haloperidol decanoate shortage in Canada. Schizophr Bull. 2017;43(Suppl 1): S201–S202.

[107] American Society of Health-System Pharmacists. Current drug shortages. Accessed April 14, 2025. https://www.ashp.org/drug-shortages/current-shortages.

