# Choosing among the long-acting injectable antipsychotics: an evidence-based pragmatic guide – ERRATUM

**DOI:** 10.1017/S109285292510059X

**Published:** 2025-11-04

**Authors:** Leslie Citrome

Cambridge University Press apologises for an error in the originally published article. The CME/CE Information was not originally included in the article PDF. This has now been updated.
